# *In Vitro* Cytotoxicity of Fluorescent Silica Nanoparticles Hybridized with Aggregation-Induced Emission Luminogens for Living Cell Imaging

**DOI:** 10.3390/ijms14011080

**Published:** 2013-01-07

**Authors:** Yun Xia, Min Li, Tao Peng, Weijie Zhang, Jun Xiong, Qinggang Hu, Zifang Song, Qichang Zheng

**Affiliations:** 1 Department of General Surgery, Tongji Hospital, Tongji Medical College, Huazhong University of Science and Technology, Wuhan 430030, China; E-Mail: xiayun7373@126.com; 2 Department of Hepatobiliary Surgery, Union Hospital, Tongji Medical College, Huazhong University of Science and Technology, Wuhan 430022, China; E-Mails: li_min@smail.hust.edu.cn (M.L.); kingtao913@163.com (T.P.); wj539@163.com (W.Z.); oldxiong@163.com (J.X.); huqg@hotmail.com (Q.H.); bioszf@163.com (Z.S.)

**Keywords:** aggregation-induced emission, fluorescent silica nanoparticles, cytotoxicity, cell imaging, silole

## Abstract

Fluorescent silica nanoparticles (FSNPs) can provide high-intensity and photostable fluorescent signals as a probe for biomedical analysis. In this study, FSNPs hybridized with aggregation-induced emission (AIE) luminogens (namely FSNP-SD) were successfully fabricated by a surfactant-free sol-gel method. The FSNP-SD were spherical, monodisperse and uniform in size, with an average diameter of approximately 100 nm, and emitted strong fluorescence at the peak of 490 nm. The FSNP-SD selectively stained the cytoplasmic regions and were distributed in the cytoplasm. Moreover, they can stay inside cells, enabling the tacking of cells over a long period of time. The intracellular vesicles and multinucleated cells were increase gradually with the rise of FSNP-SD concentration. Both cell viability and survival only lost less than 20% when the cells were exposed to the high concentration of 100 μg/mL FSNP-SD. Additionally, the cell apoptosis and intracellular ROS assay indicated that FSNP-SD had no significant toxic effects at the maximum working concentration of 80 μg/mL. This study demonstrated that the FSNP-SD are promising biocompatible fluorescent probes for living cell imaging.

## 1. Introduction

Organic dye molecules have widely been used as fluorescent probes for imaging in cell biology. However, the fluorescent intensity of the conventional organic dye molecules gets weaker due to the notorious aggregation-caused quenching (ACQ) effect when the small fluorophore molecules accumulate on the surfaces of the biomacromolecules or form nanoaggregates, for instance, clustering in the hydrophobic cavities or pockets of the folding structure [1]. In 2001, Tang *et al.* discovered a series of silole molecules which are non-luminescent in solution state but emissive in the aggregated state [2]. The new phenomenon was denominated as aggregation-induced emission (AIE) and the restriction of intramolecular rotation (RIR) was identified as a main mechanism for the AIE effect [3,4]. On the basis of RIR, a series of luminogens with various emission colors were synthesized through covalent conjugation of various functional groups to the AIE fluorophores [1].

In order to protect organic dye molecules from being destroyed by oxygen molecules in the surrounding aqueous environment and improve the signal-to-noise ratio when measuring fluorescent signals, fluorescent silica nanoparticles (FSNPs) which are loaded with hundreds of fluorescent dye molecules, receive strong interest in biolabeling application, especially various cancer cell imagings [5–7]. Besides, FSNPs also possess other advantages, such as good photostability, strong brightness, water dispersibility, easy modification and various fluorescent colors, *etc*. [8–10]. Recently, ultrasmall silica nanoparticles were approved for their first investigational new drug application, a human clinical trial launched by Memorial Sloan-Kettering Cancer Center’s Nanotechnology Center and Cornell University, by the US Food and Drug Administration (FDA). Among fluorescent nanoparticles, semiconductor quantum dots (QDs) are also a hot topic for living cell imaging, *in vivo* imaging, and diagnostics [11–13], but the cytotoxicity and potential interference of QDs should not be ignored. For cell biology and tumor imaging, a high concentration of QDs is often required [14]. The release of Cd^2+^ and Se^2+^ ions in both core and core-shell QDs can be observed in many cases [15,16]. Moreover, the process for improving the hydrophilicity of QDs is complicated [17]. In contrast, FSNPs are biocompatible and hydrophilic, which have presented a promising alternative to QDs [18]. However, since the fluorescent dye molecules are in aggregated state as a dye-doped core, the fluorescent intensity of conventional organic dye molecules is generally weak and cannot be enhanced by increasing the loading dose of the dyes, owing to the ACQ effect [19–22]. Fortunately, the AIE-active luminogens emit stronger fluorescence as a core with increased loading. However, a major concern that has arisen is whether the FSNPs hybridized with AIE luminogens cause toxic effects in living systems.

Herein, FSNPs doped with silole derivative (FSNP-SD) were fabricated through a surfactant-free sol-gel method. The emission spectra, morphology and size of FSNP-SD were examined and the FSNP-SD was utilized to stain living cell imaging. The distribution of FSNP-SD in cells was observed using transmission electron microscope (TEM). To verify whether silica nanoparticles doped with AIE molecules could be applied to living cell imaging in future, the potential toxicity of FSNP-SD, including cell morphological change, cell viability, cell survival, cell apoptosis and intracellular reactive oxygen species (ROS), was investigated in both tumor cells and normal cells.

## 2. Results and Discussion

### 2.1. Fabrication and Characterization of FSNP-SD

**Figure 1:**
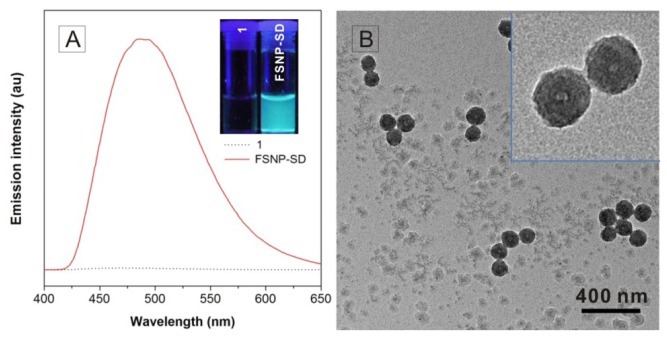
Characterization of FSNP-SD. (**A**) The emission spectra of **1** and FSNP-SD in ethanol solutions. Excitation wavelength: 371 nm. Inset: photograph of **1** and FSBP-SD in ethanol solutions taken under 365 nm UV irradiation from a hand-held lamp; (**B**) TEM images of FSNP-SD at different magnifications.

The FSNPs loaded with silole derivative molecules were fabricated though surfactant-free sol-gel method (Scheme I), according to the reported literature [23]. Finally, the molecule **1**, an AIE-active molecule, accumulated in the core of silica network. In this study, **1** is chemically bound to the network-structured SiO_2_, therefore the luminogens do not leak out of the nanoparticles. The emission spectra of **1** and FSNP-SD in ethanol solutions were measured (Figure 1A). The fluorescent signal was scarcely detectable when **1** was dissolved in ethanol solution due to active intramolecular rotation. On the contrary, the suspension of FSNP-SD emitted strong fluorescence at the peak of 490 nm, contributing to stern restriction of the intramolecular rotation of **1** by the silica network. Similarly, when the suspension of FSNP-SD was taken upon irradiation with an UV lamp of 365 nm, stronger fluorescence was visible than that of **1** in ethanol solution. The TEM images indicate that all the nanoparticles are spherical, monodisperse and uniform in size, with an average diameter of approximately 100 nm (Figure 1B).

### 2.2. Cell Imaging

**Figure 2:**
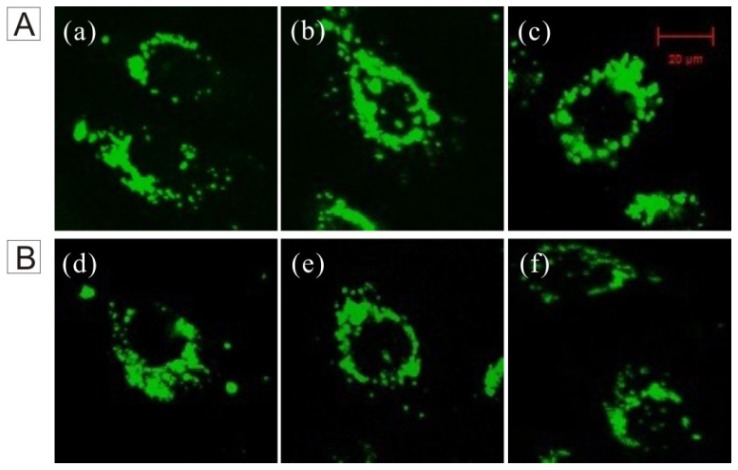
Fluorescence images of HeLa cells stained with FSNP-SD. (**A**) The cells stained with various concentrations of FSNP-SD (**a**) 20; (**b**) 40 and (**c**) 60 μg/mL for 4 h; (**B**) Duration of intracellular fluorescence after 40 μg/mL FSNP-SD treatment: (**d**) 1 day; (**e**) 3 days and (**f**) 5 days. 10 μg/mL is equal to 1 μmol/L luminogens. Scale bar = 20 μm.

The FSNP-SD possesses a good fluorescent property in the spectrofluorometer test. Would FSNP-SD show excellent performance to living cell imaging? To test this, the imaging was investigated using HeLa cells for fluorescent imaging. As shown in Figure 2, the FSNP-SD stained the cytoplasmic regions emitting green fluorescence, but no fluorescence was observed in the cell nucleus, which reduces the potential effect on the metabolism of nucleic acid. The fluorescent intensity of the cells treated with FSNP-SD was increased with an increase of the nanoparticles concentration (Figure 2A), suggesting that the cell imaging efficiency of FSNP-SD is dose-dependent. Moreover, HeLa cells still sustained green fluorescence after several days, even five days after incubation with 40 μg/mL of FSNP-SD (Figure 2B). Evidently, the FSNP-SD can remain inside cells for a long period of time, thus enabling long-term living cell tracking.

### 2.3. Distribution of FSNP-SD in Cells

**Figure 3:**
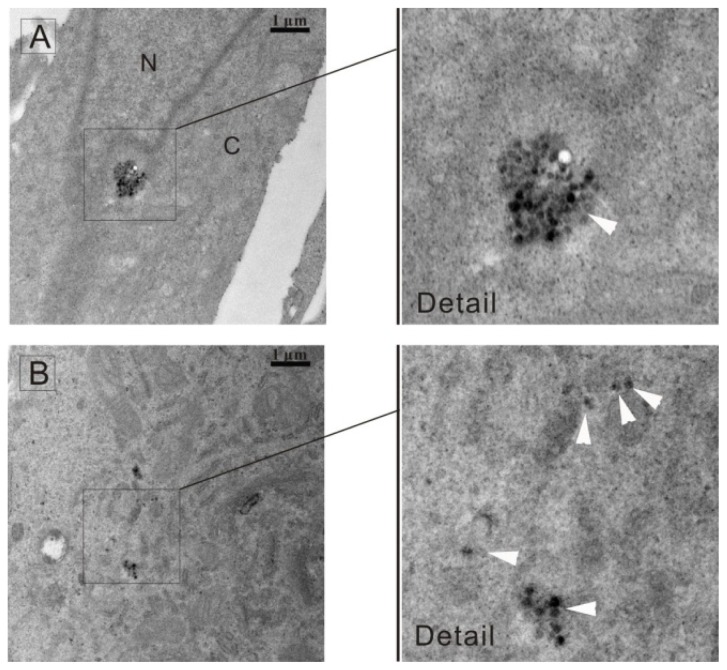
TEM images of HeLa cells treated with FSNP-SD. The nanoparticles located in the cytoplasm with (**A**) agglomerate (**B**) scattered states. Arrowheads indicate the nanoparticles. N: nucleus; C: cytoplasm. Scale bar = 1 μm.

The uptake of the silica nanoparticles is through endocytosis [24,25], which is a process that cells capture extracellular molecules in vesicles derived from the plasma membrane [26]. The nanoparticles were then released and distributed in the cytoplasm. To clarify the distribution of FSNP-SD in cells, TEM analysis of HeLa cells treated with 40 μg/mL of nanoparticles for 24 h were performed. It was observed that the nanoparticles located in the cytoplasm but not inside the nucleus and the nanoparticles were present in the form of agglomerate (Figure 3A) or scatter (Figure 3B), which was consistent with the fluorescent images in Figure 2.

### 2.4. Effect of FSNP-SD on Cellular Morphology

**Figure 4:**
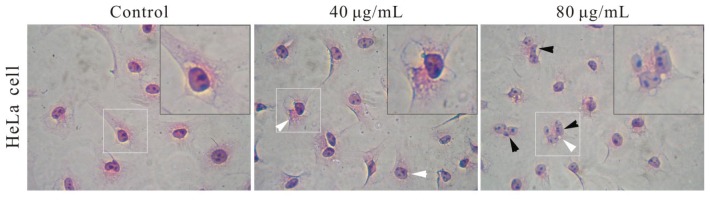

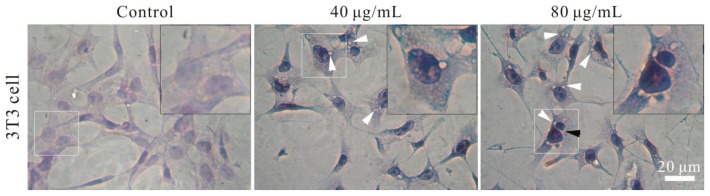
Morphological changes of HeLa cells and 3T3 cells after exposure to various concentrations of FSNP-SD for 48 h. White arrowhead: intracellular vesicles. Black arrowhead: multinucleated cells. Inset: magnified image of cells. Scale bar =20 μm.

To confirm whether or not these nanoparticles can be further applied in living cell imaging, the cytotoxicity of FSNP-SD and their precursors must be examined. Silica, the network-structured shell of FSNP-SD, has been developed as an important matrix of nanomaterial. Compared with FSNPs, semiconductor quantum dots have potential cytotoxicity due to leaching heavy metals [27]. In previous study, AIE molecules have no significant toxic effects on cell proliferation [28,29]. However, currently, there is limited knowledge about the relative assessments of FSNP hybridized with AIE molecules. To begin with, the effect of FSNP-SD on cellular morphology was observed to reflect cell injuries in Figure 4. Compared with the control group, the cell shape and chromatin condensation had no obvious changes, but the intracellular vesicles or endosomes and multinucleated cells increased gradually with the increase of FSNP-SD concentrations. The vesicles also confirm that the FSNP-SD enter into cells through endocytosis, but the mechanism of multinucleation, perhaps attributed to cell fusion and karyokinesis without cytokinesis, is ambiguous on nanotoxicology research [30,31].

### 2.5. Cell Viability and Survival

**Figure 5:**
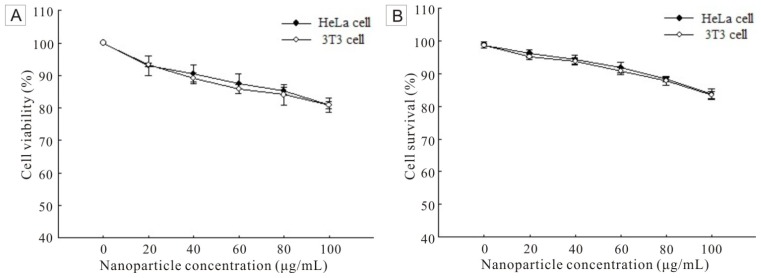
The cell viability and survival of HeLa cells and 3T3 cells after exposure to FSNP-SD for 48 h.

Mitochondrial dehydrogenase, a significant parameter of cell viability, can convert the tetrazolium component (3-(4,5-dimethylthiazol-2-yl)-2,5-diphenyltetrazolium bromide, MTT) into a formazan product [32]. The absorbance of formazan at 490 nm is proportional to the amount of living cells. Therefore, the FSNP-SD toxicity at cellular level was examined by using MTT assay (Figure 5A). The cell viability was determined against the negative control (0 μg/mL). As increasing the concentration of FSNP-SD, the cell viability, both HeLa cells and 3T3 cells, decreased. However, up to the high concentration of 100 μg/mL FSNP-SD, the viability of HeLa cells and 3T3 cells was 80.85% and 80.90%, respectively. To verify the result of MTT assay, a trypan blue exclusion assay was further performed. Trypan blue is a membrane-impermeable dye, which is not taken up by living cells with an intact plasma membrane, but included by dead cells with a leaky membrane. The result showed that the cell survival rate was related with the concentration of FSNP-SD (Figure 5B). As the nanoparticle concentration increased, the cell survival rate decreased. When the nanoparticle concentration was increased to 100 μg/mL, the percentage of survival cells was reduced to 83.77% and 83.49% for HeLa cells and 3T3 cells, respectively.

### 2.6. Cell Apoptosis Induced by FSNP-SD

**Figure 6:**
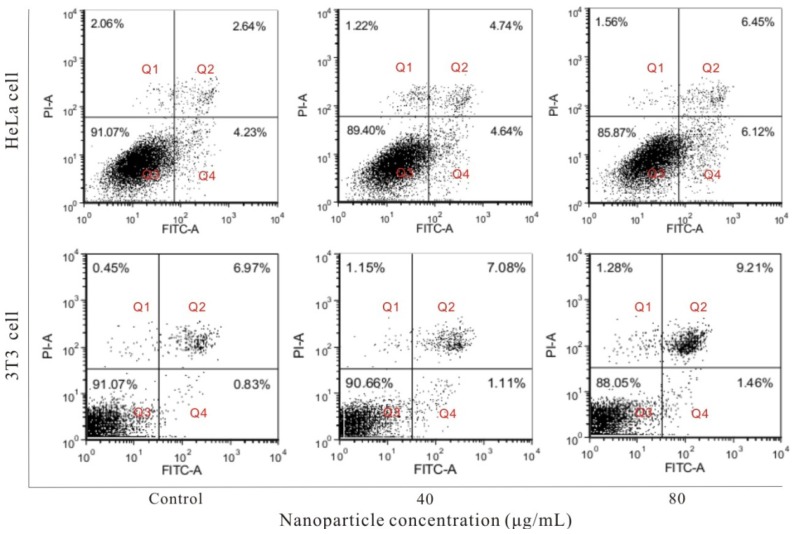
The effects of FSNP-SD on the cell apoptosis. Hela cells and 3T3 cells were treated with FSNP-SD for 48 h and detected with annexin V-FITC/PI double staining by flow cytometry.

Cell apoptosis is also an important parameter for toxicity of nanoparticles. To explore whether the FSNP-SD can cause cell damage through accelerating apoptosis, the apoptotic cells were detected by using Annexin V-FITC/PI double staining (Figure 6). The cells stained with Annexin V-FITC alone demonstrate early apoptosis (Q4), while they labeled with Annexin V-FITC and PI represent late apoptosis (Q2). The number of apoptotic cells includes all the early and late apoptotic cells. Compared with the negative control group, after treatment of HeLa cells with 40 and 80 μg/mL FSNP-SD for 48 h, the apoptosis rate was (9.38 ± 2.91)% and (12.57 ± 3.51)%, respectively. Similarly, the apoptosis rate of 3T3 cells was (8.19 ± 5.88)% and (10.67 ± 5.24)%, respectively. These results indicate that FSNP-SD have no discernible impact on cell apoptosis at maximum working concentrations.

### 2.7. Generation of Intracellular ROS

**Figure 7:**
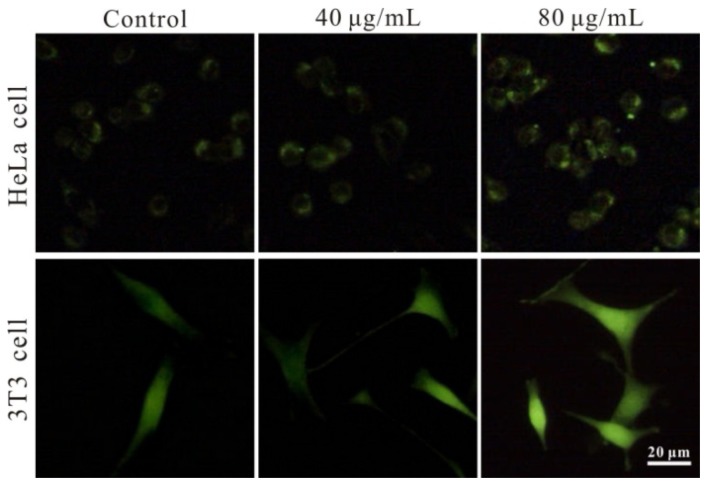
Effects of FSNP-SD on the level of ROS in HeLa cells and 3T3 cells. The cells were treated with 0 (control group), 40 and 80 μg/mL FSNP-SD for 24 h. The cells were stained with DCFH-DA and observed under a fluorescent microscope. Scale bar = 20 μm.

Intracellular ROS level reflects toxic influence of nanoparticles to living cell imaging, which can damage the function of mitochrondia, triggering the cell apoptosis process [33]. Herein, the level of intracellular ROS was determined in HeLa cells and 3T3 cells treated with 0, 40 and 80 μg/mL FSNP-SD, respectively. The fluorescent intensity of DCF, an indication of oxidative stress in suffered cells, was increased in a dose-dependent manner after 24 h exposure to the nanoparticles (Figure 7). However, even though the concentration of nanoparticles increased to 80 μg/mL, the fluorescent intensity was intensified indistinctively, suggesting that FSNP-SD induce oxidative stress.

## 3. Experimental Section

### 3.1. Materials

The precursor 1,1-dimethyl-2,5-bis[4-(2-bromoethoxy)phenyl]-3,4-diphenylsilole (**2**) was provided by Prof. Ben Zhong Tang from Hong Kong University of Science and Technology. Tetraethoxysilane (TEOS), (3-aminopropyl)triethoxysilane (APS), dimethylsulfoxide (DMSO) and other reagents were all purchased from Aldrich and used as received. Culture medium and fetal bovine serum (FBS) were purchased from Hyclone Labs. (Logan, UT, USA). Annexin V-FITC Apoptosis Detection Kit was obtained from KeyGEN BioTECH (Nanjing, China). 3-(4,5-dimethylthiazol-2-yl)-2,5-diphenyltetrazolium bromide (MTT), trypan blue exclusion assay kit, trypsin and penicillin-streptomycin mixture were bought from Sigma-Aldrich (St. Louis, MO, USA). 2,7-dichlorohydrofluoroscein diacetate (DCFH-DA), hematoxylin and eosin stain assay kit was got from Beyotime Institute of Biotechnology (Jiangsu, China).

### 3.2. Fabrication and Characterization of FSNP-SD

The FSNP-SD was prepared by a surfactant-free sol-gel method in a one-pot experimental procedure described previously [23]. Silole-APS compound (**1**) was synthesized by stirring a mixture of 6 μmol of **2** and 16 μmol of APS in 50 mL DMSO overnight and then **1** was added into a mixture of 64 mL ethanol, 1.28 mL ammonium and 7.8 mL distilled water. The solution was stirred for 1 h to prepare the silole-silica cores at room temperature. After slowly adding a mixture of 2 mL TEOS and 8 mL ethanol, the mixture was stirred at room temperature for 3 h to coat the nanocores with silica shells. The nanoparticles were rinsed three times with ethanol solution and dispersed in 1× PBS for cell experiments.

The emission spectra of molecule **1** and FSNP-SD in ethanol solution were collected using a Perkin-Elmer LS55 spectrofluorometer (Perkin-Elmer Corporation, Waltham, MA, USA) with an excitation wavelength of 371 nm. The sample was loaded into a quartz cell for measurement. Similarly, **1** and suspension of FSNP-SD in ethanol solution were illuminated with an UV lamp. The morphology and size of FSNP-SD were observed under a Tecnai G2 20 TEM (FEI Corporation, Eindhoven, The Netherlands) operated at 200 kV. The samples were prepared by placing a drop of FSNP-SD in absolute ethyl alcohol onto a copper grid and stained negatively with 2% phosphtungstic acid. The average nanoparticle size was measured by considering 100 particles into account.

### 3.3. Cell Culture and Imaging

HeLa cervical cancer cells and NIH 3T3 mouse fibroblast cells, purchased from Cell Resource Center, Shanghai Institutes for Biological Sciences, Chinese Academy of Sciences (Shanghai, China), were cultured in minimum essential medium (MEM) and RPMI 1640 medium, respectively, supplemented with 10% (*v*/*v*) FBS and 1% (*v*/*v*) penicillin-streptomycin (100 U/mL penicillin G and 100 μg/mL streptomycin) in a 5% CO_2_, 90% relative humidity incubator at 37 °C.

HeLa cells were grown overnight on a round cover slip mounted onto a 6-well plate. The living cells were incubated with 1500 μL per well of 40 μg/mL FSNP-SD and passaged at 70% to 80% confluence. The fluorescent intensity of cells treated with FSNP-SD was observed at different time points of first day, third day and fifth day. The cells were washed three times with 1× PBS and imaged under a Zeizz LSM 510 META two-photon microscope system (Carl Zeizz, Jena, Germany).

### 3.4. Transmission Electron Microscope

The distribution of nanoparticles in HeLa cells was further evaluated using TEM. Briefly, the cells were incubated with 40 μg/mL FSNP-SD for 24 h and fixed with 2.5% glutaraldehyde buffered in 1× PBS for 2 h at room temperature. Then, the cells were washed three times with 1× PBS, collected in centrifuge tubes and post-fixed with 1% osmium tetroxide for 2 h. Fixed cells were washed three times again with 1× PBS, and dehydrated through a series of alcohol concentrations (40%, 50%, 70%, 80%, 90%, 95% and 100% ethanol). The cells were finally treated with propylene oxide followed by 1:1 propylene oxide and resin overnight to evaporate the propylene oxide. The cells were subsequently embedded in araldite resin and then ultra-thin sections were cut using glass knives. They were then stained with lead nitrate and analyzed under Tecnai G2 20 TEM (FEI Corporation, Eindhoven, The Netherlands) at 200 kV.

### 3.5. Observation of Cellular Morphology

The cellular morphology was observed using the hematoxylin and eosin staining (HE staining). Hela cells and 3T3 cells were plated into a 12-well plate overnight. Following treatment described in previous section for 48 h, the cells were fixed with 4% paraformldehyde, stained with hematoxylin and eosin to improve visualization. Then, the cells were observed under an Olympus CKX41 phase contrast microscope (Olympus, Tokyo, Japan) to detect morphological changes such as cellular shape, chromatin condensation, intracellular vesicles and multinucleated cells.

### 3.6. Cell Viability Assay

Cell viability was assessed by mitochondrial dehydrogenase activity using MTT assay according to the manufacturer’s instruction. Briefly, 3 × 10^3^ cells were incubated per well in 200 μL of medium in a 96-well plate overnight. Then, fresh medium containing various concentrations of FSNP-SD was added into the plate. After treatment for 48 h, 20 μL MTT solution (5 mg/mL in PBS) was added into each well. In this period, the living cells converted the tetrazolium component into a formazan product. After incubation for 4 h at 37 °C, 100 μL DMSO was added to dissolve the formazan product. After 10 min, the absorbance (*A*) readings at 490 nm were measured using a 96-well plate reader (Thermo, Waltham, MA, USA). Every experiment was performed in triplicate.

### 3.7. Trypan Blue Exclusion Assay

To determine the number of viable cells in a cell suspension, trypan blue exclusion assay was further performed. Briefly, the cells were plated at the density of 5 × 10^3^ cells per well in 24-well plate overnight. Then, the cells were treated with fresh culture medium containing various concentrations of FSNP-SD for 48 h. Finally, the cells were harvested by trypsinization and scored as alive or dead using trypan blue according to the manufacturer’s instructions. Cell numbers were determined using a conventional hemocytometer. All determinations were performed in triplicate.

### 3.8. Cell Apoptosis Assay

Cell apoptosis was detected by using Annexin V-FITC Apoptosis Detection Kit according to the modified manufacturer’s protocol. HeLa cells and 3T3 cells were grown in cell culture medium containing various concentrations of FSNP-SD for 48 h. Then the apoptosis effect was determined by using flow cytometry. Briefly, adherent and floating cell populations were combined, washed twice with 1× PBS, and then resuspended in 300 μL of binding buffer at a density of 2 × 10^5^ cells/mL. Afterwards, the cells were treated with 5 μL Annexin V-FITC and 5 μL propidium iodide (PI) over 10 min at room temperature in the dark and an immediate analysis was performed with a FACSCalibur cytometry (BD Biosciences, San Jose, CA, USA). For each sample, 1 × 10^4^ cells were measured. Every experiment was performed in triplicate.

### 3.9. ROS Measurement

The production of intracellular ROS was measured using 2,7-dichlorohydrofluoroscein diacetate (DCFH-DA). DCFH-DA passively enters the cells and then reacts with ROS to form a highly fluorescent compound dichlorofluorescein (DCF). The cells were plated onto a 6-well plate overnight and treated with various concentrations of the nanoparticles for 24 h. After incubating with 10 μM DCFH-DA solution for 20 min at 37 °C, the cells were washed three times with 1× PBS. The fluorescence of DCF in cells was observed under an Olympus BX41 inverted fluorescence microscope (Olympus, Tokyo, Japan) with excitation wavelength of 488 nm and the imaging of the cells was captured using a digital CCD camera. The experiment was performed three times.

## 4. Conclusions

In summary, silica nanoparticles hybridized with silole derivative were successfully fabricated by the surfactant-free sol-gel method. The FSNP-SD were spherical in shape, uniform in size and monodispersed. The precursor silole derivative **1** was non-emissive in ethanol solution, but the FSNP-SD emitted strong green fluorescence upon photoexcitation because the intramolecular rotation of **1** was blocked by the silica-network. The nanoparticles selectively stain cytoplasm and can stay inside cells for a long period of time, even up to five days. Moreover, the cytotoxicity of FSNP-SD was investigated including morphological changes, cell viability, cell survival, cell apoptosis and intracellular ROS, suggesting that they possesses low toxicity in HeLa cells and 3T3 cells. In further work, we will conjugate specific biomolecules such as antibodies, folic acid, biotin and glycyrrhetinic acid to the surface of FSNP-SD, to improve its targeting specificities to cancer cells. Therefore, the FSNP-SD are promising biocompatible fluorescent probes for live cancer cell imaging.

## Figures and Tables

**Scheme I:**
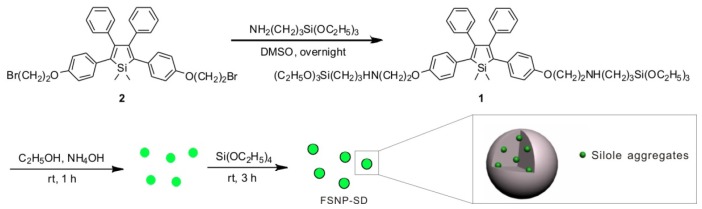
Fabrication of FSNP-SD via surfactant-free sol-gel method.
